# Behavioural side effects of inhaled corticosteroids among children and adolescents with asthma

**DOI:** 10.1186/s12931-022-02112-8

**Published:** 2022-07-28

**Authors:** Karoline S. Bodum, Britta E. Hjerrild, Søren Dalsgaard, Sune L. M. Rubak

**Affiliations:** 1 Department of Psychiatry, Aalborg University Hospital, Mølleparkvej 10, 9000 Aalborg, Denmark; 2 National Center for Register-Based Research, Aarhus University, Aarhus, Denmark; 3 Danish Center for Pediatric Pulmonology and Allergology, Department of Paediatrics and Adolescents Medicine, Aarhus University Hospital, Aarhus, Denmark

**Keywords:** Asthma treatment, Paediatrics, Adverse drug events, Inhaled corticosteroids, Behavioural changes, Pharmacovigilance, Underreporting

## Abstract

**Background:**

Inhalation corticosteroids (ICS) are prescribed for treatment of asthma in approximately 3% of all children in Denmark. Despite limited evidence, case reports suggest that ICS-related behavioural adverse drug events (ADEs) may be frequent. In general, underreporting of ADEs to official databases is common, and little is known about doctor’s clinical experiences with behavioural ADEs when prescribing ICS for children with asthma. The objective was to investigate the extent of behavioural ADEs in children with asthma treated with ICS by comparing database findings to experiences of specialist doctors.

**Methods:**

First, databases of the European Medicines Agency (EMA) and the Danish Medicines Agency (DKMA) were searched for reports made by healthcare professionals about behavioural ADEs in children from 2009 to 2018. Second, questionnaire data on behavioural ADEs were collected from eight of the 11 specialist doctors responsible for treating children with asthma at the six paediatric departments in Central Denmark Region and North Denmark Region.

**Results:**

EMA and DKMA had registered 104 and 3 reports, respectively, on behavioural ADEs during the 10-year study period. In contrast, five of the eight specialist doctors (45.5%) had experienced patients who had developed behavioural changes during ICS treatment. However, none of the five specialist doctors had filed reports on these events to DKMA.

**Conclusion:**

Behaviour-related ADEs to ICS in children with asthma are likely to be highly underreported in official databases and doctors treating children with ICS should be aware of potential ADEs and consider submitting ADE reports whenever appropriate.

**Supplementary Information:**

The online version contains supplementary material available at 10.1186/s12931-022-02112-8.

## Background

In Denmark, asthma is one of the most common chronic diseases in children with a prevalence of 10% at the age of 15 years [1]. Inhaled corticosteroids (ICS) effectively reduce asthma symptoms [2–5]. In 2018, approximately 3% of all Danish children were prescribed ICS [6]. Oral administration of steroids is spread systemically in the body and is known to be associated with several behavioural changes, including mood changes, hyperactivity, and inattention [7]. However, it is less documented to which extent such behavioural changes also applies to ICS [8], although case-reports have been published [7, 9, 10]. Attention deficit/hyperactivity disorder (ADHD) is a prevalent neurodevelopmental disorder characterised by symptoms of inattention and/or hyperactivity and is also associated with a 45–53% increased risk of asthma [11], possibly due to a mix of environmental and genetic risk factors [12].

The specialist doctors in charge of treating children and adolescents with asthma have a special obligation to make report if a patient experiences behavioural disturbances presumably caused by treatment with ICS [13]. In Denmark, adverse drug events (ADEs) are reported to the Danish Medicines Agency (DKMA) [13], which notifies the EudraVigilance (EV) database run by the European Medicines Agency (EMA). EMA collects information about ADEs from clinical trials and European national health authorities to ensure proper pharmacovigilance [14]. Across drug groups, a relatively high proportion of ADEs are unfortunately not reported to the relevant authorities [15]. Thus, databases ensuring pharmacovigilance may not give an accurate account of the occurrence of behavioural disturbances, including those related to ICS. The consequence of underreporting of adverse drug events is that the DKMA/EMA cannot properly evaluate and monitor drug safety. The objective of this study was to investigate the extent of behavioural ADEs in children treated with ICS, as experienced by the treating specialist doctor. Furthermore, the study aimed to evaluate the possibility of underreporting by the treating specialist doctor by comparing database findings and the experiences of these specialists on the reporting ADEs.

## Methods

We included data on behavioural ADEs from the official DKMA/EMA databases over a 10-year period from 2009 to 2018, namely the official EMA/DKMA databases and questionnaire data from specialist doctors treating children with asthma at regional hospitals in Denmark.

### Questionnaire data

A questionnaire was designed to evaluate the experiences of paediatric doctors in charge of treating children and adolescents with asthma concerning behavioural ADEs presumably caused by ICS treatment (see Additional file 1: Appendix). The questionnaire included items concerning the treating specialist’s overall clinical experience as well as own knowledge of the extent of behavioural changes in relation to ICS treatment. Another item covered the specialist’s clinical experience with this type of ADE. Finally, the questionnaire included items on the doctors’ attitude towards informing about and reporting such ADEs. In total, the questionnaire consisted of nine items. Three of these questions were only relevant for those who had ever suspected behavioural side effects of ICS in their patients. Throughout the study, the doctors were asked to provide responses to different types of answer categories, including a Likert scale from 1 to 10. The questionnaire could either be completed online using SurveyMonkey [16] or on paper.

Within Central Denmark Region and North Denmark Region there are six paediatric hospital departments. The head of each department agreed for the department to participate in the study and helped identify all specialists responsible for treating children with ICS at that department. An email including an introduction letter, a PDF document with the questionnaire and a link to SurveyMonkey was sent to the head of each of the six departments, who then forwarded it to the identified doctors and asked them to fill in the questionnaire (N = 11). The questionnaire data was collected in 2019, just prior to COVID-19.

### Database search strategy

European and National Danish data on reported ADEs were obtained from freely accessible online databases at the EMA [17] and DKMA [18] official websites, respectively. The present study included ADEs reported for the relevant ICS drugs defined by the Anatomical Therapeutic Chemical Classification System (ATC), specifically the ATC-code R03BA (beclomethasone, budesonide, ciclesonide, fluticasone and mometasone) [19]. We did not include ADEs reported for combination products to avoid inclusion of ADEs which might be caused by other drug-classes than corticosteroids.

Due to differences between the interfaces of the EMA and DKMA websites, it was not possible to do completely parallel searches. In EMA, the following criteria were chosen: Gender (male, female, not specified), age group (0–1 month, 2 months – 2 years, 3–11 years and 12–17 years), gateway date (2009–2018), reporter group (healthcare professional), seriousness (serious, non-serious, not available), reaction groups (psychiatric disorders) and geographic origin (European Economic Area). In DKMA, the following criteria were chosen: Gender (male, female, unknown), age group (0–9 years, 10–19 years), time period (2009–2018), reporter group (healthcare professional), administration route (inhalation), severity (cases of death, serious, not serious) and organ class (psychiatric disturbances). Since it was impossible to filter for administration form in the EMA database an additional search was made in the DKMA database including all administration forms.

## Results

### Questionnaire

Among the 11 invited specialists, eight filled in the whole questionnaire (72.2%). The participating doctors represented five of the six included departments and did not differ from non-participating doctors in terms of age, gender, or years of work experience. The department from which no questionnaires were collected was one of the smallest of the six. The average number of years of experience among the specialists in paediatrics was 7.94 (range from 0 to 27), and they reported being responsible for treating a mean of 871 patients with asthma per year (range 50 to 3000).

On average, the specialists estimated that they had experienced or suspected behavioural ADEs in 4% (SD = 3.8) of the children and adolescents treated with ICS, based on experiences from the last 100 patients they had treated. Five doctors had treated one or more patients who they experienced had developed behaviour-related side effects of ICS and estimated that among the last 100 patients treated with ICS, a mean of 2.6% (SD = 3.4) were suspected to have one or more of these behavioural ADEs (Table 1).

**Table 1:** Physicians’ answers—extent of behaviour related ADEs

Question	Answer	
Based on your own experiences how would you estimate the extent of behavioural changes (mood changes, hyperactivity, irritability, aggressiveness, etc.) as side effect to treatment with ICS? *State an approximate percentage based on experiences from the last 100 children you have treated*	4% (SD = 3.8) (0–10%)	
Have one or more of your patients experienced side effects of ICS in the form of behavioural changes such as mood changes, irritability, aggressiveness, etc.?	Yes	No
	5	3
If yes, how big a proportion of the last 100 children you have treated with ICS, do you suspect have one or more of this type of side effect? *If you answered “no” in question 2, you can skip this*	2.6% (SD = 3.4) (0–8%)	

The numbers displayed in “yes” and “no” reflect the number of doctors who chose each option of answers to the question. The numbers 4% and 2.6% are averages of the doctors’ responses. Ranges are displayed in brackets

None of the participating specialists had filed an official report to the DKMA on a suspected behavioural ADE to ICS, although half of the participating doctors had answered in the questionnaire that they had changed ICS treatment due to behavioural side effect. When the doctors were asked to rate the likelihood on a Likert scale rate that they would make a report to the DKMA if they ever suspect ADEs to ICS treatment in a patient (1 = not likely, 10 = 100% likely), the average number answered was 4.63 (SD = 3.38) (Table 2).

**Table 2:** Doctors’ answers—attitude towards reporting of ADEs

N (total) = 8			
Question	Answer		
Have you reported this side effect to the DKMA database?	Yes	No	Not relevant
	–	6	2
What is the likelihood that you would make a report if you suspect one of you patients experiencing side effects of ICS in the form of behavioural changes such as mood changes, irritability, aggressiveness, etc.? *1* = *no likelihood, 10* = *100% likelihood*	4.63 (SD = 3.38) (1–10)		

The numbers displayed in “yes”, “no” and “not relevant” reflect the numbers of doctors who chose each option of answers to the question. The number 4.57 is an average of the eight participating doctors’ responses to the question. Ranges are displayed in brackets

Finally, the doctors were asked if they ever discussed potential behavioural changes to ICS treatment with their patients and/or their parents The doctors were asked to rate their answer on a scale from one to 10 (one = never, 10 = always); the average rate to this question was and 4.57 (SD = 4.23).

### Data from EMA

**Fig. 1:**
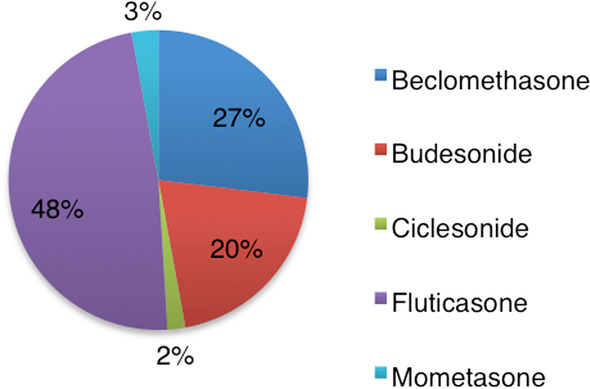
Distribution of reports in the EV database concerning the R03BA drugs

Between 2009 and 2018, a total of 104 reports about psychiatric side effects of the ICS drugs were reported to the EMA database. Reports regarding fluticasone accounted for the majority of reports (48.0%), and the fewest reports concerned ciclesonide and mometasone, which constituted only 1.9% and 2.9% of the reports, respectively (Fig. 1).

**Fig. 2:**
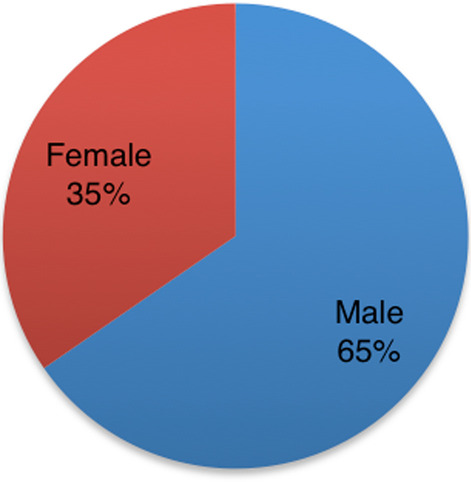
Distribution of reports in the EV database regarding males and females

In relation to gender, the majority of reports concerned males (65.4%) (Fig. 2).

**Fig. 3:**
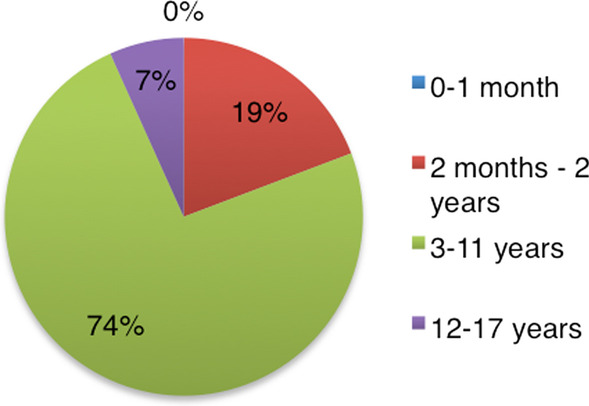
Distribution of reports in the EV database regarding the different age groups

Most of the reports were on children aged three to 11 (74.0%), and children under age three (19%), whereas 7% of the reports concerned ages 12 to 17 years (Table 3, Fig. 3).

**Table 3:** Characteristics of behavioural ADEs to ICS in EMA from 2009 to 2018

	Number of reports	Proportion of all reports [%]
N (total) = 104		
*Drug*		
Beclomethasone	28	26.92
Budesonide	21	20.9
Ciclesonide	2	1.92
Fluticasone	50	48.08
Mometasone	3	2.89
*Sex*		
Male	68	65.38
Female	36	34.62
*Age*		
0–1 month	–	–
2 months–2 years	20	19.23
3–11 years	77	74.04
12–17 years	7	6.73

Characteristics on accumulated reports made by healthcare professionals in the European Economic Area from 2009 to 2018 found in the EudraVigilance database

Number of reports per year ranged from five in 2011 to 18 in 2017 (Table 4).

**Table 4:** Annual reports (EMA)

	2009	2010	2011	2012	2013	2014	2015	2016	2017	2018	Total:
Beclomethasone	–	1	3	2	1	2	3	2	7	7	28
Budesonide	2	3	–	1	3	3	4	1	2	2	21
Ciclesonide	–	–	1	–	–	–	–	–	1	–	2
Fluticasone	4	3	1	4	4	6	7	6	8	7	50
Mometasone	1	–	–	–	–	1	–	–	–	1	3
In total, all ICS	7	7	5	7	8	12	14	9	18	17	104

Number of annual reports about psychiatric ADEs found in the EudraVigilace database from 2009–2018 regarding children and adolescents treated with either beclomathasone, budesonide, ciclesonide, fluticasone or mometasone

### Data from DKMA

Reports on behavioural ADEs by Danish healthcare professionals over a 10-year period from 2009 to 2018 resulted in three reports. Two of these were on budesonide in 2015 and 2016 (Table 5).

**Table 5:** Annual reports of behavioural ADEs to ICS in Denmark

	2009	2010	2011	2012	2013	2014	2015	2016	2017	2018	Total:
Beclomethasone	–	–	–	–	-–	–	–	–	–	–	–
Budesonide	–	–	–	–	–	–	1	1	–	–	2
Ciclesonide	–	–	–	-	-	–	–	–	-–	–	–
Fluticasone	–	–	–	–	–	-	1	–	-	–	1
Mometasone	–	–	–	–	–	–	–	–	–	–	–
In total, all ICS	–	–	–	–	–	–	2	1	–	–	3

Number of annual reports about psychiatric ADEs to the Danish Medicines Agency from 2009–2018 regarding children and adolescents treated with either beclomathasone, budesonide, ciclesonide, fluticasone or mometasone

Both reports concerned males, and one of them was between 0 and 9 years of age, and the other between 10 and 19 years. The report on fluticasone was made in 2015 and concerned a male patient between 0 and 9 years (Table 6).

**Table 6:** Characteristics of behavioural ADE reports to ICS in Denmark from 2009 to 2019

	Incidence
N (total) = 3	
*Drugs*	
Beclomethasone	–
Budesonide	2
Ciclesonide	–
Fluticasone	1
Mometasone	–
*Sex*	
Male	3
Female	–
*Age*	
0–9 years	2
10–19 years	1

^a^Characteristics of reports made by healthcare professionals to the Danish Medicines Agency database of adverse drug events (DKMA). The Danish Medicines Agency has confirmed to the authors that, despite small cell sizes, reporting these public data are in accordance with all Danish regulations and EUs General Data Protection Regulation (GDPR)

Specifications of the reported behavioural side effects were ‘anxiety disorders and symptoms’, ‘depressed mood disorders and disturbances’, and ‘psychiatric disorders NEC’. When including other administration routes other than inhalation, the search resulted in three further reports on fluticasone. Two were related to nasal administration and the last was categorised as “unknown administration”.

## Discussion

### Main findings

The study investigated the number of filed reports on behaviour-related ADEs over a 10-year period in children and adolescents treated with ICS and compared it to experiences of specialist doctors. The primary finding was that only three reports have been submitted to the DKMA during the 10-year study period 2009–2018. A majority of experienced paediatric specialist doctors responsible for the treatment of children with asthma in two Danish geographical regions reported that they had suspected that one or more of their patients developed behavioural changes due to ICS treatment. The study showed that the experienced doctors found that they should have reported behavioural side effects of ICS treatment in 2.57% of the last 100 children with asthma they had treated. The specialists estimated the total extent of behavioural side effects of ICS treatment to be 4% on average. A report from the Danish Health Authority states that 10% of the 15-year-olds in Denmark are diagnosed with asthma [1]. If in fact 4% of these patients develop behavioural ADEs caused by ICS as estimated by the doctors in our study, this would correspond more than 5000 children and adolescents [1, 20]. Hence, behavioural ADEs due to ICS treatment in children and adolescents with asthma may be underreported. This correlates with earlier studies suggesting that ADEs in general are underreported [15].

In the period 2009–2018, 40–45,000 children and adolescents in Denmark received ICS treatment daily with primarily budesonide and fluticasone. Of the three reports made on behavioural ADEs in DKMA, two concerned budesonide and one fluticasone [6]. These two drugs are the most commonly used ICS drugs. However, due to the very low number of ADEs reported this might just be a coincidence. A total of 104 reports had been registered in the European database over the past ten years, and 48% concerned fluticasone. Reports about beclomethasone and budesonide accounted for 28% and 21%, respectively. As data on drug use are not available for all European countries, it is difficult to determine whether this distribution reflects the use of the various drugs. Norway, Sweden and Finland have databases similar to the Danish Statistics Denmark and may be accessed online; however, only in the Norwegian and Swedish databases is it possible to filter for age. In Norway, fluticasone was the most used ICS during 2009–2018, followed by use of budesonide and beclomethasone [21]. The top three of the most used ICS medications to treat children and adolescents with asthma in Sweden during the same period were budesonide, fluticasone and beclomethasone [22]. Unfortunately, free online access could not be found for databases of the remaining countries in the European Economic Area. However, a few publications have been made about ICS use among children with and without asthma in different European countries [23–26]. These studies included data regarding drug use related to asthma treatment during the period from year 2000–2008. Again, beclomethasone, budesonide and fluticasone were the most frequently used ICS drugs for treating children with asthma [23–26]. Our results must be interpreted with caution when comparing them to the drugs most frequently represented among the reports from the EV database, because these reports were made from 2009–2018. In addition, it should be noted that all countries in the European Economic Area have contributed to the figures in the database. It remains unknown whether ADEs with behavioural changes are more frequent in fluticasone, compared with the other ICE drug types, or whether this just reflects a relatively higher use of this drug.

In this study the majority of the paediatric doctors responsible for ICS treatment reported that they had experienced one or more patients with suspected ICS-related behavioural changes, but none had ever reported such a case to the proper authorities. Furthermore, approximately half of the doctors had previously talked with the patients and/or parents about potential behavioural side effects of ICS treatment, suggesting that the doctors are aware of the occurrence of behavioural changes related to ICS use among Danish children and adolescents with asthma. To learn more about the low rate of reporting to authorities, it would be interesting to investigate the doctors’ reasons for not reporting these cases. Inch et al. published a systematic review on ADE reporting behaviour among patients and healthcare professionals, respectively, showing that reporting behaviour differed between these two groups, especially in relation to body systems affected and the therapeutic categories [27]. Another study by Klinnert et al. aimed to examine behavioural adjustment and emotion regulation among six-year-old children with asthma and compare them to healthy controls [28]. They investigated evaluations of parents, healthcare professionals and the children themselves and found that mothers of children with asthma tend to report higher Total Behaviour Problem scores and more internalizing problems than mothers of children without asthma [28]. No difference was seen in behavioural adjustment scores from healthcare professionals. This indicates that mothers of children with asthma are either more aware of or have a lower threshold for addressing behavioural changes in children with asthma compared to healthcare professionals [28]. The authors suggested that this might be due to the aberrant behavioural patterns seen in children with asthma being too subtle to reach diagnostic threshold and therefore not noted or considered as serious ADEs by professionals [28]. The findings of this study would most likely have been different if patient reporting had been included in the study [28].

### Strengths and limitations

The sample size regarding the questionnaire study of physicians in charge of asthma treatment was small, which reduces the statistical power, and our findings may be coincidental. However, the included doctors, except from one, were specialist paediatricians, primarily engaged in treating children with asthma, which made them highly qualified to participate in this survey, and increased the validity of their responses. The participation rate was moderate to good (72%), non-participation did not seem strongly skewed, and hence, the findings are likely representative for specialists responsible for treating children with asthma at paediatric departments. A second limitation is that the doctors participating in this study might have been influenced by their personal interests, which would have impaired the validity of the questionnaire data, introducing bias towards either greater or smaller likelihood of underreporting. A third limitation is that the questionnaire was not validated or tested. In hindsight, it would also have been valuable if we had collected information on the duration and dose of the prescribed corticosteroids, and on the doctors’ reasons for not reporting the adverse effects.

Furthermore, the two online databases (DKMA and EMA) were not identical regarding their search criteria. Unfortunately, the EMA database lacks the possibility to filter ADEs by administration route. This may have increased the number of reported ADEs in the EMA database relative to the number of reports in the Danish database. To investigate this further, a search was made in the DKMA database including the remaining administration routes. This search resulted in three further reports on fluticasone. Two of these were related to nasal administration, and the third was stated as unknown administration. It remains difficult to predict if filtering for administration route in the EV database would have shown a different result by making a comparison of the number of reports in the DKMA database regarding administration by inhalation with multiple routes of administration. This is because the DKMA database contains very few reports on ADEs to ICS treatment in children in general. A final limitation of this study is that the relationship between asthma and behavioural disturbances is not fully understood. Thus, the possibility of such symptoms being misinterpreted as side effect to ICS treatment should be considered. In the systematic review by McQuaid et al. the following causality mechanism between asthma and behavioural problems were addressed [29]: (1) asthma-related stressors might lead to difficulties in behavioural adjustment, (2) increased parasympathetic activity as seen in people with depressive symptoms can cause bronchoconstriction, (3) depression might influence the immune system leading to inflammation, and (4) the relationship could rely upon social factors, and that difficulties in parenting early in children’s life could influence the immune system and thereby increase the risk of developing asthma [29]. Furthermore, several epidemiological studies have found strong associations between asthma and ADHD [11]. Twin studies suggest that 68% of the phenotypic association between the hyperactive-impulsive subtype ADHD and asthma is caused by genetics [30] and genome-wide association studies suggest that asthma and ADHD are genetically correlated [31]. There is also data suggesting that the more severe asthma, the higher the risk of ADHD and this may also influence the emergence of behavioural changes seen after initiation of ICS in children with a higher genetic susceptibility for one or both disorders [12, 32]. Anxiety disorder and depression may also be more common in children with asthma, especially in those with uncontrolled asthma symptoms [33]. In short, not all behavioural changes after initiation of ICS may be true ADEs. Unrecognized mental disorders in children with asthma, including ADHD, anxiety disorder, and depression, are important differential diagnoses to ADEs. If one or more of the above-mentioned mechanisms are accountable for the relationship between behavioural changes and asthma in children, behavioural disturbances in children with asthma might not be caused by ICS treatment.

In general, few studies on the possible link between ICS treatment and behavioural changes exist and these are either case reports, cross-sectional designed or case–control studies, and findings of these studies point in different directions [9]. In a letter to the editor, Hederos described an unpublished prospective follow-up study of 60 preschool children recently diagnosed with asthma [9]. According to Hederos, they found that after 18 month of treatment with high dose (770 μg daily) budesonide, 9% had experienced more hyperactivity and aggressiveness, suggesting that ICS treatment can impact on children’s behaviour [9]. We find that these results support our thesis that behavioural side effects are more frequent than reported.

### Future clinical and research implication

The link between different systemic side effects and ICS treatment of children with asthma was reviewed in 2017 determining frequencies of ADEs associated with paediatric asthma medication and concluded both growth suppression and adrenal deficiency to be related to the use of ICS in children [34]. These findings increase the likelihood that behavioural side effects can also occur in children with asthma in ICS treatment, and this theory is supported by findings of systemic treatment with steroids causing this type of ADE [7]. Thus, further studies including double-blinded randomised controlled trials are needed to establish a possible link between ICS treatment and behavioural changes.

## Conclusions

This study supports the hypothesis that behavioural side effects of ICS treatment in children and adolescents with asthma are underreported in Denmark and the EU. Only three ADE reports have been filed to DKMA and only 104 reports to European EV databases over the past decade, despite an increasing number of children being treated with ICS. In addition, paediatric specialists within the field of asthma agree that such behavioural changes after initiation of ICS are not uncommon. The reason for not reporting has not been examined and the mechanisms behind the association between ICS treatment and behavioural changes are not yet fully understood. Thus, further studies including randomised controlled trials are needed to improve existing guidelines for treatment of children and adolescents with asthma.

## Supplementary Information

**Additional file 1:** Appendix 1.

## Data Availability

Not applicable.

## Abbreviations

ICS: Inhalation corticosteroids
ADE: Adverse drug event
EMA: European Medicines Agency
DKMA: Danish Medicines Agency
