# Supplementary material for: Behavioural side effects of inhaled corticosteroids among children and adolescents with asthma
Source: Respir Res. 2022 Jul 28;23:192. doi: 10.1186/s12931-022-02112-8 (PMC9330944; doi:10.1186/s12931-022-02112-8)
Supplement: Supplementary file 1 — Additional file 1: Appendix 1. [file 12931_2022_2112_MOESM1_ESM.pdf]

Spørgsmål vedr. adfærdsændringer hos børn og unge der får inhalationssteroid

I forbindelse med min 12. semester specialeopgave på medicinstudiet vil jeg gerne undersøge forskellige forhold vedrørende adfærdsændringer hos børn og unge som bivirkning til inhalationssteroid.

Baggrundsoplysninger:

Antal år som speciallæge i pædiatri: ________

Omtrentlige antal patienter du behandler pr. år: ________

1. Baseret på dine egne erfaringer, hvordan vil du vurdere omfanget af adfærdsændringer (humørændringer, hyperaktivitet, irritabilitet, aggressivitet, mv.) som bivirkning til behandling med inhalationssteroid?
   Angiv omtrentlige %-del. Svaret ønskes baseret på dine erfaringer fra de sidste 100 børn, du har behandlet.

   ________ %

2. Har en/flere af din(e) patienter oplevet bivirkninger til inhalationssteroid i form af adfærdsændringer såsom humørændringer, hyperaktivitet, irritabilitet, aggressivitet, mv.?

   ☐ Ja

   ☐ Nej

3. Hvis ja, hvor stor en %-del af de seneste 100 børn, du har behandlet med inhalationssteroid, anslår du, har en eller flere bivirkninger inden for denne kategori?
   Såfremt du har svaret "nej" i spørgsmål 2, bedes du se bort dette spørgsmål.

   ________ %

4. Har du da indberettet denne bivirkning til Lægemiddelstyrelsens bivirkningsdatabase?

☐ Ja

☐ Nej

☐ Har ikke haft patienter med denne type bivirkning

5. Har du oplevet at ændre en patients behandling på baggrund af adfærdsbivirkninger til inhalationssteroid?

☐ Ja

☐ Nej

☐ Har ikke haft patienter med denne type bivirkning

6. Hvor stor er sandsynligheden for, at du vil indberette til Lægemiddelstyrelsen, hvis du mistænker, en af dine patienter oplever bivirkninger til inhalationssteroid i form af adfærdsændringer såsom humørændringer, hyperaktivitet, irritabilitet, aggressivitet, mv.?

1 = ingen sandsynlig, 10 = 100 % sandsynlighed

1	2	3	4	5	6	7	8	9	10

7. Hvor ofte har du talt med dine patienter eller disses forældre om adfærdsændringer som mulig bivirkning til behandling med inhalationssteroid?

1 = aldrig, 10 = altid

1	2	3	4	5	6	7	8	9	10

https://da.surveymonkey.com/r/SZ2QS9M
